# 3D multi-physics uncertainty quantification using physics-based machine learning

**DOI:** 10.1038/s41598-022-21739-7

**Published:** 2022-10-19

**Authors:** Denise Degen, Mauro Cacace, Florian Wellmann

**Affiliations:** 1 RWTH Aachen University, Computational Geoscience, Geothermics and Reservoir Geophysics (CGGR), Mathieustraße 30, 52074 Aachen, Germany; 2 GFZ German Research Centre for Geosciences, Telegrafenberg, 14473 Potsdam, Germany; 3 Fraunhofer Research Institution for Energy Infrastructures and Geothermal Systems (IEG), Am Hochschulcampus 1, 44801 Bochum, Germany

**Keywords:** Solid Earth sciences, Mathematics and computing

## Abstract

Quantitative predictions of the physical state of the Earth’s subsurface are routinely based on numerical solutions of complex coupled partial differential equations together with estimates of the uncertainties in the material parameters. The resulting high-dimensional problems are computationally prohibitive even for state-of-the-art solver solutions. In this study, we introduce a hybrid physics-based machine learning technique, the non-intrusive reduced basis method, to construct reliable, scalable, and interpretable surrogate models. Our approach, to combine physical process models with data-driven machine learning techniques, allows us to overcome limitations specific to each individual component, and it enables us to carry out probabilistic analyses, such as global sensitivity studies and uncertainty quantification for real-case non-linearly coupled physical problems. It additionally provides orders of magnitude computational gain, while maintaining an accuracy higher than measurement errors. Although in this study we use a thermo-hydro-mechanical reservoir application to illustrate these features, all the theory described is equally valid and applicable to a wider range of geoscientific applications.

## Introduction

Physics-based models, meaning models that are governed by partial differential equations, are extensively used in geosciences^1–6^. These models are all based on well-established physical principles, which provide a high-fidelity approximation of the Earth’s system dynamics. Typical problems in geosciences rely on a large number of parameters in order to describe the multidimensional character of the underlying processes in both space and time^1–6^. Parameter estimation is often based on limited data, which, together with our incomplete knowledge of the heterogeneous physics at play, degrades the performance of currently adopted solutions^7^. The challenge here is to understand both the structure of the prediction system and to quantify its uncertainty^8^. Uncertainty quantification (UQ) requires to develop a confidence metric to measure predictions, their validation against available data, and their sensitivity upon variations in the parameter space. This prerequisite often results in high-dimensional problems, which become computationally intractable if based on traditional probabilistic methods such as Markov chain Monte Carlo^8^. Any advancement in UQ analyses requires to improve upon traditional statistical error analysis. A common strategy is to rely on surrogate models, constructed either for the entire state^9,10^ or limited to the observation space alone^11–13^. Projection based surrogate models for the entire state, as constructed in Hesthaven et al.^9^ and Benner et al.^10^, are based on the physical model, and share the advantage of preserving the underlying physics. However, these models are limited in their range of applications, e.g., they can not generally be applied to hyperbolic and nonlinear problems as typically encountered in geoscientific applications^10,14^. Data-driven surrogate models for the observation space alone are not applicable for nonlinear problems either^15^. In addition, these models do not preserve the physics^11–13^.

Recent advances in statistical and machine learning (ML) methods, together with increased computational resources offer a new opportunity to expand our knowledge about the system Earth from available data^16–18^. Several free computational ML libraries (e.g., PyTorch^19^, TensorFlow^20^) are nowadays available to allow automatic management and integration of the steadily increasing stream of geospatial data. However, the requirement of a reliable and robust surrogate model^18^ has so far limited a straightforward application of ML techniques for UQ analysis in geosciences.

A commonly used method for combining physics and data-driven techniques is the physical-informed neural network (PINN)^21–25^. However, as we will demonstrate later in this study, PINNs likely underperform for applications as those targeted in our study, and we further elaborate on current disadvantages of PINNs as presented in Chuang and Barba^26^, and Wang et al.^27^. In this study, we propose an alternative method, namely, the non-intrusive reduced basis (NI-RB) method, a hybrid physics-based machine learning technique^14,18,28^, for the construction of reliable and robust surrogate models. By combining physics-based modeling with data-driven concepts it extends the range of applicability of each individual method, provides substantial computational gain with negligible degradation in the model accuracy, and enables the application of UQ for real case 3D multi-physics simulations. In addition, its theoretical formulation is independent of the specific physics at play, and therefore the NI-RB is applicable to a wide range of geoscientific applications.

## Construction and evaluation of the surrogate model

**Figure 1:**
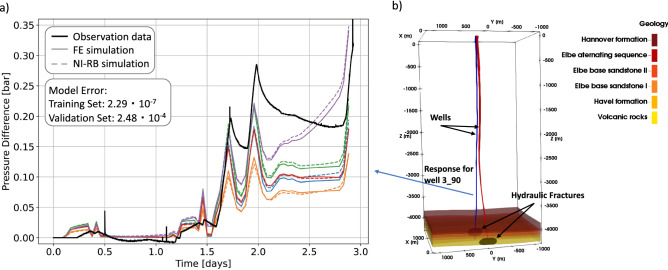
(**a**) Representation of the accuracy of the surrogate model of Groß Schönebeck for five different parameter realizations of the validation data set. The solid black line denotes the observation data, the colored solid lines the FE solutions and the dashed lines the RB solutions. (**b**) Overview of the geological reservoir model of Groß Schönebeck. Next to the six geological units also the two wells and the hydraulic fractures are indicated.

To illustrate the applicability of the NI-RB method, we selected a real-case reservoir problem: a hydrofracture treatment performed at the geothermal research facility of Groß Schönebeck in northern Germany^29^, Fig. 1. The targeted problem exhibits all features of interest, that is, high dimensionality (heterogeneous distribution of material properties) and tightly coupled multi-physics (fluid dynamics coupled to non-linear solid thermo-mechanics). For the model validation, we rely on matching the pore pressure response at the monitoring well E GrSk 3_90 (marked in blue in Fig. 1b), which we consider as representative of the far-field response over the entire reservoir to the hydraulic stimulation at the opposite well (500 m distance)^30^. The evolution in time of the monitored over-pressure (Fig. 1a) displays a rather complex pattern, with local rapid changes superimposed on a monotonic long-term diffusive trend. This is an important aspect since deep learning algorithms would perform poorly in matching such a complex time series (with the amount of data at hand) as they would for example fail to predict the timing and wavelength of the rapid bursts in the system response^27^. Instead, we are able to replicate the pressure evolution curve both with respect to the long-term and short-term characteristics. The constructed surrogate model provides an accuracy of 2.29 × $10^{-7}$ for the dimensional training set, and 2.48 × $10^{-4}$ for the dimensional validation set, and it requires five basis functions for its construction. For the estimation of the accuracy the mean squared error between the full and reduced solutions has been calculated. Deviations between measurements (solid black curve in Fig. 1a) and the FE solutions (colored solid curves in Fig. 1a) are instead in the order of 10^−1^, being caused by the input fracture model (see also the “Uncertainty quantification” Section). Our surrogate model, therefore, provides a higher accuracy than the accuracy of the original model, and we can consider the approximation errors from the surrogate model as negligible.

So far, we have limited our discussion to global values, that is we have been interested in the accuracy of the RB model for the entire training and validation data set. In an attempt to understand the time evolution in the error datasets, we investigate five simulations that we chose randomly from the validation dataset. For all five realizations, we obtain maxima in the errors within the time period between 2.1 and 2.8 days, which is exactly during the time window that features the highest variability in the model response. However, also for this time period, the introduced errors are smaller than the deviations between the FE solutions and the observation data. We consider this as proof that the NI-RB method is suited for the construction of reliable surrogate models for tightly coupled multi-physics problems even for systems that showcase a rather complex and variable response in time and space.

## Global sensitivity analysis

**Figure 2:**
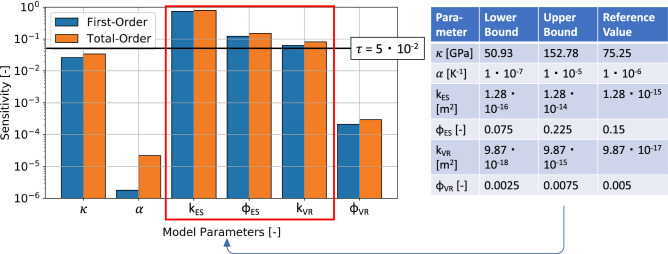
Global sensitivity analysis for the model of Groß Schönebeck with a threshold value of $5 \times 10^{-2}$, as well as the lower and upper bounds presented on the right side. Note that $\kappa$ denotes the solid bulk modulus, $\alpha$ the thermal expansion coefficient, *k* the permeability, $\phi$ the porosity, and the subscripts *ES* and *VR* the Elbe sandstone I layer, and the Volcanic rocks, respectively.

In order to identify the model parameters having the highest influence on the resulting pressure evolution, we carry out a global sensitivity analysis. For our problem we allow six parameters to vary, that is, the solid bulk modulus, the thermal expansion coefficient, the permeability and the porosity of the Elbe base sandstone I and the Volcanic rocks, the latter being the reservoir layers targeted during the hydraulic stimulation^29,30^. In Fig. 2 we list all parameters with their range of variations used in the sensitivity analysis. The goal of the sensitivity analysis is to identify the parameters that minimize the misfit between the observation data and the modeling results the most. To this end, we carry out our sensitivity analysis with respect to the mean squared difference between simulated and measured data. Given the error of the surrogate models, we consider a threshold value of $5 \times 10^{-2}$ for the tolerance in our analysis.

We found that the parameters that exert the highest influence on the system response are the permeability and the porosity of the Elbe base sandstone I and the permeability of the underlying volcanic rocks, with the sandstone permeability exerting the highest influence and that of the Volcanics the lowest influence. We also note that the obtained difference between the first- and total-order contributions are negligibly small for all these three parameters and that consequently, we have only a minor parameter correlation.

We can further observe that thermal effects (quantified by the impact of assumed variations in the thermal expansion coefficient) do not affect the pressure distribution significantly and that also the mechanical parameter considered (solid bulk modulus) only has a minor influence. Hence, the thermo-elastic stress transfer is not impacting the pressure response, which is also confirmed by a study matching the microseismicity following the treatment^31^.

## Uncertainty quantification

**Figure 3:**
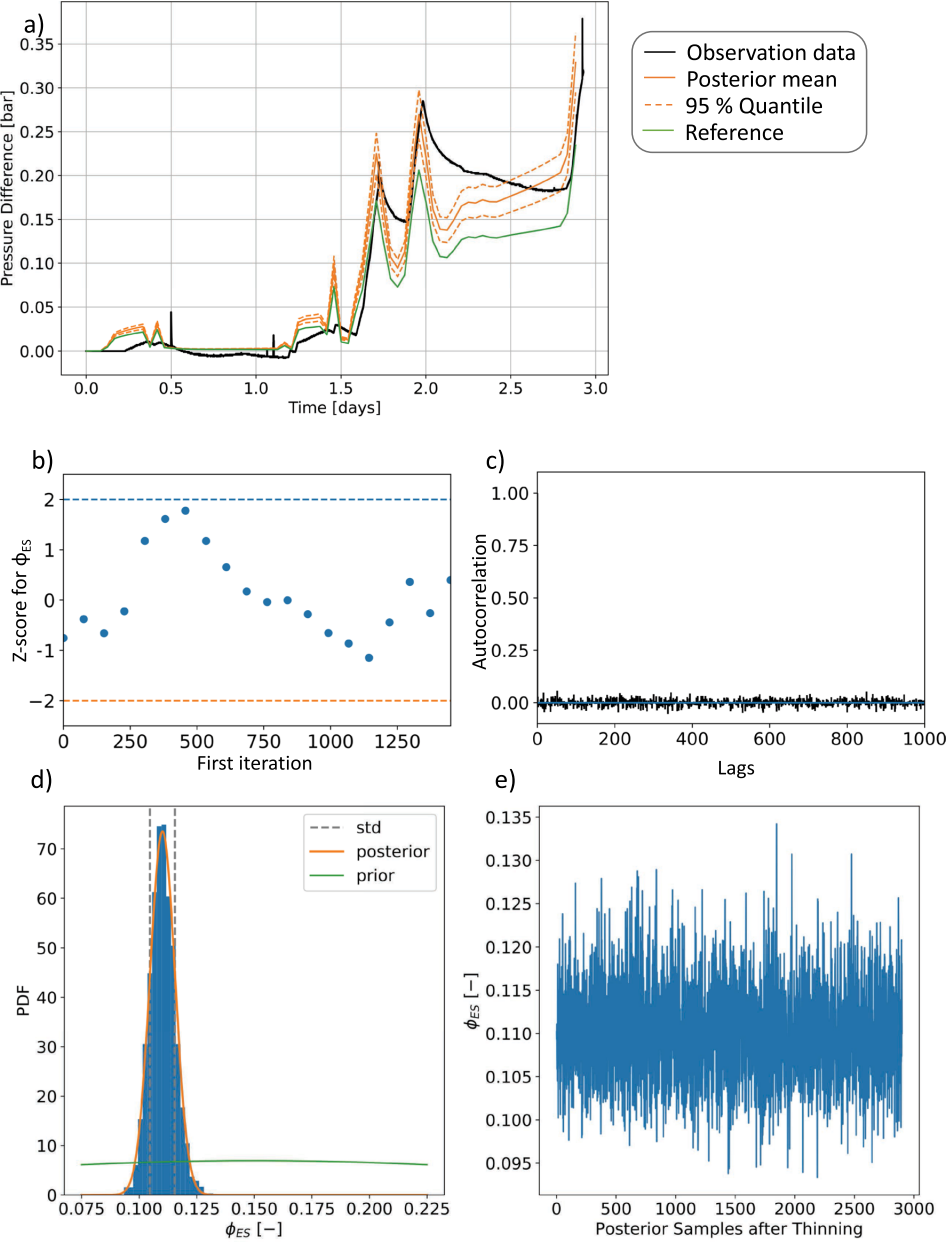
Posterior analysis of pressure response and exemplary of the porosity of the Elbe base sandstone I ($\phi _{ES}$). Shown are the (**a**) pressure distribution for the well 3_90 (both posterior mean and 95 % quantile), (**b**) Geweke Plot, (**c**) autocorrelation, (**d**) posterior parameter distributions, and (**e**) the trace.

The global sensitivity analysis has led to a reduction of the dimension of the parameter space, and accordingly of the sampling ensemble required for UQ. Therefore, we focus our UQ on the resulting three most influencing parameters; the permeability and porosity of the Elbe base sandstone I layer and the permeability of the Volcanics. All other parameters are kept constant, according to their reference values (Fig. 2). We start our UQ based on the pressure response to the stimulation at the well 3_90 as computed before and after the stochastic model calibration. The results are summarized in Fig. 3a. The green curve represents the “trial-and-error” calibration obtained by a previous work^30^, which we take in this study as the mean of the prior. The solid orange curve is our posterior mean, while the dashed orange curve is the 95 % quantile of the pressure. Comparing the simulations to the measurements, we note the presence of a source of epistemic uncertainties, which is resulting from the input fracture model used for the modeling. This overestimates the fracture closure after each stimulation stage, visible as the systematic overshooting of the modeled pressure relaxation after each pressure peak during each cycle. However, this source of uncertainty does not impact any results presented in this manuscript.

In Fig. 3b–e, we show exemplarily the posterior analysis for the porosity of the Elbe base sandstone I. The complete information for all remaining parameters is provided in the Supplementary Material.

We observe a shift in the posterior mean of the permeability and a significant reduction in the uncertainty. This indicates that incorporating observation data yields a reduction in uncertainties. Before discussing the results further, we inspect the robustness of the MCMC run. A commonly applied measure of ergodicity of the chain is the Geweke diagnostic test^32^. In this test, variance and mean values for multiple segments of the chain are compared. If the measures are comparable, then this is an indication for a stationary chain. Results of this test are shown in 3b). In our cases, they do not exceed a range between − 2 to + 2 indicating stability. Also, the autocorrelation (3c) shows robustness by having values close to zero. Lastly, we observe a suitable mixing in the trace (3c). This can be visually seen by having a different porosity value for each sample. A non-suitable mixing would be visible by adjacent samples having the same porosity values. In this case, we would have rejected samples indicating an improper choice of the proposal step size. With the here presented approach, we can demonstrate an efficient and robust UQ workflow, even for this complex non-linear physical simulation. Note that the execution of the UQ requires about 20 min for 300,000 iterations. As a comparison the same analysis using the full model would have taken more than 50 compute-years, already accounting for a possible parallelization on high-performance infrastructures. Except for the large reduction in computation time, we observe that the UQ greatly reduces the uncertainties of the input parameters, as seen in the posterior distribution of the parameters and the small variation ranges in the distribution of the pressure response. For the UQ, the usage of a physics-based ML approach has clear advantages since we preserve the main physical characteristics and use the ML technique for the projection step only. Using either purely data-driven approaches or other physics-based methods, such as physics-informed neural networks (PINNs), yields degradations of the UQ analysis, especially when considering noisy data^33^. This is why approaches such as BPINNs have been developed^33^, which overcome this issue but have the disadvantage of including the hyperparameters in the UQ analysis, which results in a loss of the physical meaning of the system. In contrast, the non-intrusive RB method results in surrogate models that can be used in a flexible way for common parameter estimation methods, SAs, and UQ.

## Discussion

In this paper, we presented the non-intrusive RB method, a hybrid physics-based machine learning method that enables the construction of robust, scalable, and interpretable surrogate models for a wide range of geoscientific applications where multiple simulations are required. To showcase the potential of the method, we use the geothermal study of Groß Schönebeck because it compromises the typical challenges of geoscientific applications: being high-dimensional and nonlinear, and considers a tightly coupled multi-physics setting. For this model, we reduce the computational time from about 1.5 h (already using parallelization on High-Performance infrastructures) to about 3 ms (desktop computer). This reduction in computation time is a result of an enormous reduction in the dimensionality from 278,095 degrees of freedom to only five, yielding a speed-up of six orders of magnitude.

In contrast to classical machine learning or PINNs, the non-intrusive RB method does not treat physics simply as data or as a constrain. The POD step performed as a first step extracts the characteristical physical behavior and the machine learning technique is used to determine the weight of these characteristics only. This has the advantage that (i) the amount of data required for the methodology is greatly reduced, and (ii) the approximation errors are mainly related to the weighting and therefore the main characteristics of the response are preserved. The aspect of data is especially important since this application shows that the data generation is costly, which holds for geoscientific applications in general. In this work, 150 samples are sufficient for the generation of the surrogate model. For data-driven approaches, we would expect the amount of data to be magnitudes higher^23^. Generally, machine learning has the disadvantage of not being rigorously proven, which is problematic for predictions and their reliability. This problem is also shared by the non-intrusive RB method, though only at the weighting step. In addition, the NI-RB is based on a rigorously proven method^9,10,34^. So, by simplifying the problem setting to an elliptic or parabolic PDE, we can always fall back on the proven methodology. Finally, the presented non-intrusive RB method constructs a map from the model parameters (e.g., rock properties) to the model response making it ideally suited for parameter estimation studies. Other physics-based machine learning methods such as PINNs are meant for state estimation problems^25^. Hence, modifications are required before being applied to parameter estimation studies.

At this stage, we would like to discuss the differences between PINNs in detail since the method shares commonalities regarding the basic idea of combining physics and data-driven approaches, but differs fundamentally from a methodological perspective to the NI-RB technique. PINNs consider the physics as a constrain in the loss function, which can be either done in the strong form^25^ or its variational counterpart^22^. In both scenarios, the crucial aspect is that the physics is treated as one of possibly many constraints allowing for solutions that no longer represent the original physical problem as presented in Chuang and Barba^26^. Whether it is true that the NI-RB method does not preserve the physics, the machine learning stage is sued only to determine the weights between the different functions used to capture the physical behaviour. Therefore the obtained deviations only affect the solutions up to a combination of scalar values. Further disadvantages of PINNs are that the solution is enforced at specific points only^25^ and that a model is constructed from these selected points to the entire state distribution for one parameter realization only. This would hinder the use of such methods for the case at hand, where we are interested in the solutions to multiple realizations and to preserve the original structure of the model, as well as in a flexible and performant use for UQ all aspects that demands to maintain the map from the material parameters to the full state response, as done via the NI-RB method. Chuang and Barba^26^ raise further the concern that PINNs are computationally inefficient, due to the usage of automatic differentiation. They also show in their computational fluid dynamics applications that the method yields performance and convergence issues. These issues might occur also for other geoscientific applications, adding on the reason behind our choice not to rely on PINNs in this work and instead present a method closer to physics-driven approaches overcoming the presented issues. Arguably some of these limitations might be compensated by using variational formulations^22,24^ or transfer learning concepts^21^. Nonetheless, both of these approaches are constructed with data-driven methods in mind. For geothermal applications, we have only very sparse data sets (and situations can become easily worse for other geoscience applications)^7,35^, these data sets would need to be enriched with numerical simulations to produce a sufficient amount of data for machine learning methods. However, the sparsity will likely yield an insufficient amount of validation data if we include the measurement data in the model construction. Therefore, we prefer a method using simulation data only and being based on physics-driven approaches as closely as possible without losing applicability in the nonlinear setting. The latter aspect is especially important in terms of predictability^18^.

To sum up, the non-intrusive RB method is well suited for the construction of surrogate models for a wide range of geoscientific applications, enabling the possibility to gain new insights into this field.

## Methods

In the following, we introduce the governing equations, and the physics-based machine learning method, namely the non-intrusive reduced basis method. We use the method to construct reliable and rigorous surrogate models, which is of utmost importance for scientific machine learning^18^. Furthermore, we briefly explain the concepts of uncertainty quantification and global sensitivity studies.

### Governing equations

For the case study of Groß Schönebeck, we consider a coupled thermo-hydro-mechanical forward problem as implemented in the software GOLEM^1^, which is based on the finite element solver MOOSE^36^. The formulation of the fluid pressure $p_f$ is derived from the fluid mass balance and can be expressed as^1^:

1 $$\frac{1}{M_b}\frac{\partial p_f}{\partial t} + \nabla \cdot q_D = 0,$$

2 $$\text {with} \quad q_D = -\frac{k}{\mu _f} (\nabla p_f - \rho _f g).$$

Here, $M_b$ denotes the Biot modulus, *t* the time, *k* the permeability, $\mu$ the dynamic viscosity, $\rho$ the density, *g* the gravitational acceleration, and the subscript *f* the fluid component.

The equation for the temperature is an expression of the energy balance, and is given by^1^:

3 $$\begin{aligned} \left( \rho c \right) _b \frac{\partial T}{\partial t} + \nabla \cdot \left( \left( \rho c\right) _f q_D T - \lambda _b \nabla T \right) = 0, \end{aligned}$$

where *c* is the specific heat capacity, *T* the temperature, $\lambda$ the thermal conductivity, and the subscript *b* denotes the bulk component.

We take the momentum balance to derive the equation for the effective stress $\sigma '$, yielding^1^:

4 $$\begin{aligned} \nabla \cdot \left( \sigma ' - \beta p_f \mathbbm {1} \right) + \rho _b g = 0. \end{aligned}$$

The Biot coefficient is denoted by $\beta$, and $\mathbbm {1}$ is the rank-two identity matrix. For further information regarding the coupling scheme, we refer to Cacace and Jacquey^1^.

### Non-intrusive reduced basis method

The field of machine learning is rapidly growing and more and more incorporated into scientific applications. This growth is also tightly linked to large open-source Python libraries such as TensorFlow and PyTorch, making the methodologies accessible in a user-friendly black-box approach^17^. Nonetheless, machine learning for scientific purposes faces major challenges, when applied in a black-box model^18,28,37^. To ensure the scientific merit, models resulting from the applied methodologies must be rigorous, reliable, scalable, generalizable, and interpretable^18,28,37^.

Machine learning methods are commonly entirely data-driven methods. This poses a major challenge for many geophysical applications that investigate subsurface processes since they face the problem of data sparsity, as common in many other physical applications. Instead of data, these applications usually have a good understanding of the governing physical processes (although subjected to uncertainties). This is the reason they classically focus on physics-based approaches^18^. However, computing these models is computational very demanding, making intensive parameter estimation problems prohibitive^35,38^. Therefore, a common procedure is to construct surrogate models. These surrogate models can be physics-based^9,10,34^ or data-driven^11,12,17,39^. We already addressed the shortcoming of data-driven approaches in our field. However, also physics-based surrogate models are problematic since they often have only limited applicability to nonlinear hyperbolic partial differential equations (PDEs). Hence, neither of the methods is suitable for the application presented here. Therefore, we propose the usage of a physics-based machine learning method, namely the non-intrusive reduced basis method^14,40^.

The non-intrusive reduced basis method (NI-RB) can be seen as a hybrid approach combining physics-based and data-driven techniques to overcome the limitations of both approaches. Another commonly used physics-based machine learning method is the physical informed neural network (PINN)^25^. Although, the principle idea behind both methods is similar the implementations differ.

The NI-RB method is a modification of the projection-based model order technique, from now on referred to as the intrusive reduced basis (RB) method. Both methods aim to significantly reduce the degrees of freedom while maintaining the input–output relationship. The intrusive version has been extensively studied in the field of mathematics^9,10,34^ and also for synthetic and real-case geophysical applications^35,38^. Furthermore, an goal-orientated error estimator for THM simulations is available^41^. However, this estimator is derived considering the conductive part of the heat transport and not in addition the advective part, as required here. Furthermore, having an estimator that focuses on a quantity of interest alone is not always desirable. Especially, when the prime interest of the study is to investigate the driving forces for the entirety of the model. Therefore, we use here an approach that is generally applicable. The following presentation focuses on the non-intrusive RB method and the differences to the intrusive method. We will not discuss the intrusive RB method in detail. For details regarding this method we refer to Hesthaven et al.^9^, Benner et al.^10^, and Quarteroni and Rozza^34^.

For the non-intrusive RB method, we take advantage of the circumstance that we can express the reduced solution as^14^:

5 $$\begin{aligned} {\textbf {u}}_{rb} \left( \varvec{\mu } \right) =\sum _{i=1}^r \theta _{rb}^{(i)}\left( \varvec{\mu } \right) \psi _i \qquad \in V_{rb}, \end{aligned}$$

where, ${\textbf {u}}_{rb}$ is the reduced solution, *r* the size of the reduced basis, $\theta _{rb}$ the reduced coefficients (later on referred to as weights), and $\psi _i$ are the basis functions that span the reduced space $V_{rb}$.

The non-intrusive RB method consists of two stages: the offline and online stage. During the offline stage, the surrogate model is constructed, involving all computational expensive steps. Note that this stage needs to be performed only once. In contrast, during the online stage, the surrogate model is used, enabling a fast and efficient computation. The offline stage consists of two steps. In the first step, the basis functions for the surrogate model are selected. This is commonly done via the proper orthogonal decomposition^14^. Here, we perform a singular value decomposition and truncate the eigenvectors after reaching a user-defined tolerance $\epsilon$. This tolerance is set to $1 \times 10^{-4}$ in our case to ensure that the errors induced by the approximation are negligible. The method aims to retrieve an optimal low-rank approximation. For the evaluation of the error, we are using an “energy” term according to the following definition^10,28^:

6 $$\begin{aligned} \frac{\sum _{i=1}^r \sigma ^2_i}{\sum _{i=1}^N \sigma ^2_i}\le \varepsilon . \end{aligned}$$

Here, $\sigma$ is the eigenvalue, and *N* is the total number of training samples.

The second step is the projection step. For the conventional intrusive RB method, we would employ a Galerkin projection (analogously to the finite element problem)^9^. In the case of the non-intrusive RB method, used here, the Galerkin projection is replaced by a machine learning method, such as Gaussian Process Regression or Neural Networks^14^. In this paper, we use a Neural Network since we solve for a nonlinear PDE, and Neural Networks are known for performing superior to Gaussian Process Regression in the case of nonlinearities^25^. In this projection step, we determine the weight of each basis function.

In contrast to PINNs, the non-intrusive RB method first limits the physical plausible range and then uses the neural network to determine the weight of the basis functions, whereas PINNs use the physics as a constraint inside the loss function of the neural network^25^. PINNs are classically employed for state estimation problems, whereas we need to construct a surrogate model for a parameter estimation problem. Hence, we need to define the mapping from the input parameter to the output solution, making the non-intrusive RB method advantageous.

An important aspect of the reliable construction of the surrogate model is the choice of the training set. This set should be representative of the entire parameter range but as small as possible since each sample in the training set corresponds to a costly finite element simulation. In this paper, we use the Latin hypercube sampling strategy^42^ to efficiently sample the given parameter space and produce a training set of 150 samples. The validation data set is computed separately and not extracted from the training set. It consists of 20 samples, generated with a random sampling strategy, where we ensure that the validation samples are not identical to any training samples.

Another important point is the preprocessing of the data. Note that for the pressure output we focus on the pressure differences, thus no additional scaling is required. The input parameters (i.e., the rock properties) are scaled with a normal score transformation.

The offline stage of the non-intrusive RB method is computationally expensive for two reasons. The first reason is the construction of the training set, which involves solving numerous expensive finite element simulations. We already explained above how intelligent sampling strategies can reduce the cost of this stage. Another reason why the offline stage is expensive is the determination of the hyperparameters of the neural network prediction step (e.g., number of layers, neurons, epochs). Especially for complex studies, this can be a time-consuming process. To reduce the cost, a preprocessing of the input (see explanation above) is essential. Additionally, the cost can be further reduced by using a Bayes optimization scheme^43^. For the given example the preprocessing and the Bayes optimization yield a satisfactory surrogate model. Note that for studies with a higher complexity, a Bayes optimization alone might not suffice to avoid unreasonable high offline times. In these cases, we advise using Bayes optimization with hyperband (BOHB) as the optimization method for the determination of the hyperparameters. We will not discuss the method in detail here. The general idea is to accelerate the convergence for the tuning of the hyperparameters and to enable parallel computations yielding a scalable approach. For further details, we refer to Falkner et al.^44^. A list of the hyperparameters used in this study is presented in Table 1.

**Table 1:** Hyperparamters of the neural network for the case study of Groß Schönebeck.

Hyperparameter	Value
Number of hidden layers	5
Number of neurons per hidden layer	15 (hl 1), 25 (hl 2), 30 (hl 3), 35 (hl 4), 15 (hl 5)
Number of epochs	40000
Learning rate	1e−3
Batch size	25
Loss function	Sigmoid
Optimizer	Adam

Note that hl denotes dense hidden layers.

### Global sensitivity analysis

In this paper, we perform a sensitivity analysis (SA) prior to the uncertainty quantification (UQ). The sensitivity analysis aims to determine which model parameters (e.g., rock properties such as permeability and porosity) influence the model response (e.g., pressure) the most. This step is crucial to eliminate non-influential model parameters prior to the uncertainty quantification to avoid stability issues of the uncertainty quantification and to reduce the cost of the UQ by reducing the number of required samples^35^.

Sensitivity analyses can be distinguished into two categories: local and global sensitivity analyses. Here, we employ a global SA. A detailed comparison between local and global SAs is found in Wainwright et al.^45^ for hydrological models and in Degen et al.^35^ for geothermal applications.

We use a global sensitivity analysis to investigate the entirety of the parameter space. Another reason for using the global SA is that we want to consider not only the influence of the parameters themselves but also their correlations. As a global SA method, we use the variance-based Sobol sensitivity analysis with a Saltelli sampler. The sensitivities are expressed as the ratio between the partial and total variance. To give an example, the first-order index describes the influence of the parameters themselves and is mathematically the ratio of the variance of the parameter and the total variance. The total-order index captures all parameter correlations in addition. Second-order indices define the correlations between two parameters, whereas higher-order indices express the correlation between multiple parameters. For detailed information regarding the Sobol method, we refer to Sobol^46^ and for detailed information regarding the sampling method, we refer to Saltelli^47^, and Saltelli et al.^48^.

In this work, we use the Python library SALib^49^ to perform the global sensitivity analysis. To reduce statistical errors we use 100,000 realizations per parameter resulting in a total number of required forward solves of 1,400,000. The entire execution of the global SA requires about 11 s for the non-intrusive RB approach. Note that we vary in total six parameters, including the permeabilities of the Elbe base sandstone I, and the Volcanics layer. The study uses an anisotropic permeability with different values of the permeability for the three main directions. We vary the dominant permeability of those two layers and keep the ratio to the other directions constant. So, the two other permeability values per layer vary dependent on the main value. For the permeabilities of the remaining layers, we keep the ratio to the permeability of the Elbe base sandstone layer fixed.

### Uncertainty quantification

In this work, we perform an uncertainty quantification for the model parameters influencing the pressure response in the monitoring well E GrSk 3_90 (for the reservoir model of Groß Schönebeck). The uncertainty quantification is both important to better determine the mean of the hydraulic parameters and their associated uncertainties. For the forward model, we consider thermal, hydraulic, and mechanical parameters. However, the prior global sensitivity analysis showed that the pressure response in the monitoring well is only sensitive to the hydraulic parameters.

As measurement data, we use the pressure data from the monitoring well E GrSk 3_90. This data has been conducted during a cyclic hydraulic treatment in August 2007^29^, and we use the data as presented in Jacquey et al.^30^. During the treatment, the pressure has been measured for about 4.5 days with a constant time step of 10 s, yielding 39,129 data points.

The UQ method is based on Bayes Theorem^50^:

7 $$\begin{aligned} P(u | y) \propto P(y | u) \ P(u). \end{aligned}$$

Here, *P*(*u*|*y*) is the posterior, defining the knowledge about the unknown variable *u* given the data *y*, *P*(*y*|*u*) is the likelihood, and *P*(*u*) is the prior (i.e., the knowledge of the variable *u* without any information about the data *y*)^50^. For the sampling from the posterior, we use the Markov chain Monte Carlo (MCMC) method as implemented in the Python library pyMC^51^. We use the MCMC method since it is a standard tool of comparison. The number of required samples could be reduced by using more efficient sampling methods such as Hamiltonian Monte Carlo^52^ but this does not change the general problem that this study is computationally demanding.

Analogously, to the non-intrusive RB method, we have to set a couple of hyperparameters. We set the number of total forward evaluations for the three influencing model parameters to 300,000. In addition, we use a thinning of 100 and 10,000 burn-in simulations. The parameters for the hydraulic properties during the MCMC run are listed in Table S1.

## Supplementary Information

Supplementary Information.

## Data Availability

All data generated or analysed during this study are included in these published articles^29,30^. The code for the NI-RB method including the data set for GroßSchönebeck is available in the Zenodo repository, [https://doi.org/10.5281/zenodo.7016427].

## References

[1] Cacace M, Jacquey AB (2017). Flexible parallel implicit modelling of coupled thermal-hydraulic-mechanical processes in fractured rocks. Solid Earth.

[2] Kohl T, Evansi K, Hopkirk R, Rybach L (1995). Coupled hydraulic, thermal and mechanical considerations for the simulation of hot dry rock reservoirs. Geothermics.

[3] O’Sullivan MJ, Pruess K, Lippmann MJ (2001). State of the art of geothermal reservoir simulation. Geothermics.

[4] Steefel C (2015). Reactive transport codes for subsurface environmental simulation. Comput. Geosci..

[5] Turcotte DL, Schubert G (2002). Geodynamics.

[6] van Zelst I (2021). 101 geodynamic modelling: How to design, carry out, and interpret numerical studies. Solid Earth Discuss..

[7] Degen D, Spooner C, Scheck-Wenderoth M, Cacace M (2021). How biased are our models? A case study of the alpine region. Geosci. Model Dev..

[8] Degen D, Veroy K, Wellmann F (2022). Uncertainty quantification for basin-scale geothermal conduction models. Sci. Rep..

[9] Hesthaven JS, Rozza G, Stamm Be (2016). Certified Reduced Basis Methods for Parametrized Partial Differential Equations (SpringerBriefs in Mathematics).

[10] Benner P, Gugercin S, Willcox K (2015). A survey of projection-based model reduction methods for parametric dynamical systems. SIAM Rev..

[11] Miao T, Lu W, Lin J, Guo J, Liu T (2019). Modeling and uncertainty analysis of seawater intrusion in coastal aquifers using a surrogate model: A case study in Longkou, China. Arab. J. Geosci..

[12] Mo S, Shi X, Lu D, Ye M, Wu J (2019). An adaptive Kriging surrogate method for efficient uncertainty quantification with an application to geological carbon sequestration modeling. Comput. Geosci..

[13] Navarro M (2018). Surrogate-based parameter inference in debris flow model. Comput. Geosci..

[14] Hesthaven JS, Ubbiali S (2018). Non-intrusive reduced order modeling of nonlinear problems using neural networks. J. Comput. Phys..

[15] Grepl MA (2012). Model order reduction of parametrized nonlinear reaction-diffusion systems. Comput. Chem. Eng..

[16] Bauer P (2021). The digital revolution of earth-system science. Nat. Comput. Sci..

[17] Bergen KJ, Johnson PA, Maarten V, Beroza GC (2019). Machine learning for data-driven discovery in solid Earth geoscience. Science.

[18] Willcox KE, Ghattas O, Heimbach P (2021). The imperative of physics-based modeling and inverse theory in computational science. Nat. Comput. Sci..

[19] Paszke A, Wallach H (2019). Pytorch: An imperative style, high-performance deep learning library. Advances in Neural Information Processing Systems.

[20] Abadi, M. *et al.*. TensorFlow: Large-scale machine learning on heterogeneous systems (2015). Software available from tensorflow.org.

[21] Goswami S, Anitescu C, Chakraborty S, Rabczuk T (2020). Transfer learning enhanced physics informed neural network for phase-field modeling of fracture. Theor. Appl. Fract. Mech..

[22] Goswami S, Yin M, Yu Y, Karniadakis GE (2022). A physics-informed variational deeponet for predicting crack path in quasi-brittle materials. Comput. Methods Appl. Mech. Eng..

[23] Haghighat E, Bekar AC, Madenci E, Juanes R (2021). A nonlocal physics-informed deep learning framework using the peridynamic differential operator. Comput. Methods Appl. Mech. Eng..

[24] Kharazmi, E., Zhang, Z. & Karniadakis, G. E. Variational physics-informed neural networks for solving partial differential equations. arXiv:1912.00873 (2019).

[25] Raissi M, Perdikaris P, Karniadakis GE (2019). Physics-informed neural networks: A deep learning framework for solving forward and inverse problems involving nonlinear partial differential equations. J. Comput. Phys..

[26] Chuang, P.-Y. & Barba, L. A. Experience report of physics-informed neural networks in fluid simulations: pitfalls and frustration. arXiv:2205.14249 (2022).

[27] Wang S, Yu X, Perdikaris P (2022). When and why pinns fail to train: A neural tangent kernel perspective. J. Comput. Phys..

[28] Swischuk R, Mainini L, Peherstorfer B, Willcox K (2019). Projection-based model reduction: Formulations for physics-based machine learning. Comput. Fluids.

[29] Zimmermann, G., Moeck, I. & Blöcher, G. Cyclic waterfrac stimulation to develop an enhanced geothermal system (egs)–conceptual design and experimental results. *Geothermics* **39**, 59–69. 10.1016/j.geothermics.2009.10.003 (2010).

[30] Jacquey AB (2018). Far field poroelastic response of geothermal reservoirs to hydraulic stimulation treatment: Theory and application at the groß schönebeck geothermal research facility. Int. J. Rock Mech. Min. Sci..

[31] Cacace M, Hofmann H, Shapiro SA (2021). Projecting seismicity induced by complex alterations of underground stresses with applications to geothermal systems. Sci. Rep..

[32] Geweke J (1992). Evaluating the accuracy of sampling-based approaches to the calculations of posterior moments. Bayesian Stat..

[33] Yang L, Meng X, Karniadakis GE (2020). B-pinns: Bayesian physics-informed neural networks for forward and inverse pde problems with noisy data. J. Comput. Phys..

[34] Quarteroni A, Rozza Ge (2014). Reduced order methods for modeling and computational reduction.

[35] Degen D (2021). Global sensitivity analysis to optimize basin-scale conductive model calibration-A case study from the Upper Rhine Graben. Geothermics.

[36] Permann CJ (2020). MOOSE: Enabling massively parallel multiphysics simulation. SoftwareX.

[37] Baker N (2019). Workshop report on basic research needs for scientific machine learning: Core technologies for artificial intelligence. Tech. Rep..

[38] Degen D, Veroy K, Wellmann F (2020). Certified reduced basis method in geosciences. Comput. Geosci..

[39] Lu, H., Ermakova, D., Wainwright, H. M., Zheng, L. & Tartakovsky, D. M. Data-informed emulators for multi-physics simulations. arXiv:2012.15488 (2020).

[40] Wang Q, Hesthaven JS, Ray D (2019). Non-intrusive reduced order modeling of unsteady flows using artificial neural networks with application to a combustion problem. J. Comput. Phys..

[41] Larion Y, Zlotnik S, Massart TJ, Díez P (2020). Building a certified reduced basis for coupled thermo-hydro-mechanical systems with goal-oriented error estimation. Comput. Mech..

[42] Iman, R. L. Latin hypercube sampling. Encyclopedia of quantitative risk analysis and assessment **3** (2008).

[43] Snoek J, Larochelle H, Adams RP (2012). Practical Bayesian optimization of machine learning algorithms. Adv. Neural Inf. Process. Syst..

[44] Falkner, S., Klein, A. & Hutter, F. Bohb: Robust and efficient hyperparameter optimization at scale. In *International Conference on Machine Learning*, 1437–1446 (PMLR, 2018).

[45] Wainwright HM, Finsterle S, Jung Y, Zhou Q, Birkholzer JT (2014). Making sense of global sensitivity analyses. Comput. Geosci..

[46] Sobol IM (2001). Global sensitivity indices for nonlinear mathematical models and their Monte Carlo estimates. Math. Comput. Simul..

[47] Saltelli A (2002). Making best use of model evaluations to compute sensitivity indices. Comput. Phys. Commun..

[48] Saltelli A (2010). Variance based sensitivity analysis of model output. Design and estimator for the total sensitivity index. Comput. Phys. Commun..

[49] Herman J, Usher W (2017). Salib: An open-source python library for sensitivity analysis. J. Open Source Softw..

[50] Iglesias, M. & Stuart, A. M. *Inverse Problems and Uncertainty Quantification*. SIAM News 2–3 (2014).

[51] Patil A, Huard D, Fonnesbeck CJ (2010). PyMC: Bayesian stochastic modelling in Python. J. Stat. Softw..

[52] Betancourt, M. A conceptual introduction to hamiltonian monte carlo. arXiv:1701.02434 (2017).

