# Supplementary material for: 3D multi-physics uncertainty quantification using physics-based machine learning
Source: Sci Rep. 2022 Oct 19;12:17491. doi: 10.1038/s41598-022-21739-7 (PMC9582207; doi:10.1038/s41598-022-21739-7)
Supplement: Supplementary file 1 — Supplementary Information. [file 41598_2022_21739_MOESM1_ESM.pdf]

Supporting Information for “3D Multi-Physics Uncertainty Quantification using Physics-Based Machine Learning”

Contents of this file

1. Table S1
2. Figures S1 to S3

INTRODUCTION

This supporting material provides additional information regarding the values of the hydraulic parameters before, during, and after the uncertainty quantification, which we present in Table S1. The posterior analysis for the permeability of the Elbe sandstone I layer and the Volcanic rocks are presented in Figures S1 and S2, respectively. Furthermore, we provide the Quantile-Quantile plots for all three hydraulic parameters considered as uncertain during the MCMC runin Figure S3.

TABLE S1: HYDRAULIC PROPERTIES FOR THE UNCERTAINTY QUANTIFICATION

Table S1. Properties for the model parameters during the MCMC run. Listed are the prior mean, the prior standard deviation and the proposal standard deviation. Note that k denotes the permeability, ϕ the porosity, and the subscripts ES and VR the Elbe sandstone I layer, and the Volcanic rocks, respectively.

Property	Prior Mean	Posterior Mean	Prior std	95 % Quantile	Proposal std
k_{ES} [m^2]	$1.28 \cdot ^{-15}$	$1.42 \cdot ^{-15}$	$8.00 \cdot ^{-15}$	$1.55 \cdot ^{-15}$	$3.63 \cdot ^{-14}$
k_{VR} [m^2]	$9.87 \cdot ^{-17}$	$2.15 \cdot ^{-16}$	$3.14 \cdot ^{-15}$	$5.15 \cdot ^{-16}$	$5.85 \cdot ^{-16}$
ϕ_{ES} [-]	0.15	0.11	0.15	0.12	0.59

FIGURES S1 - S3:

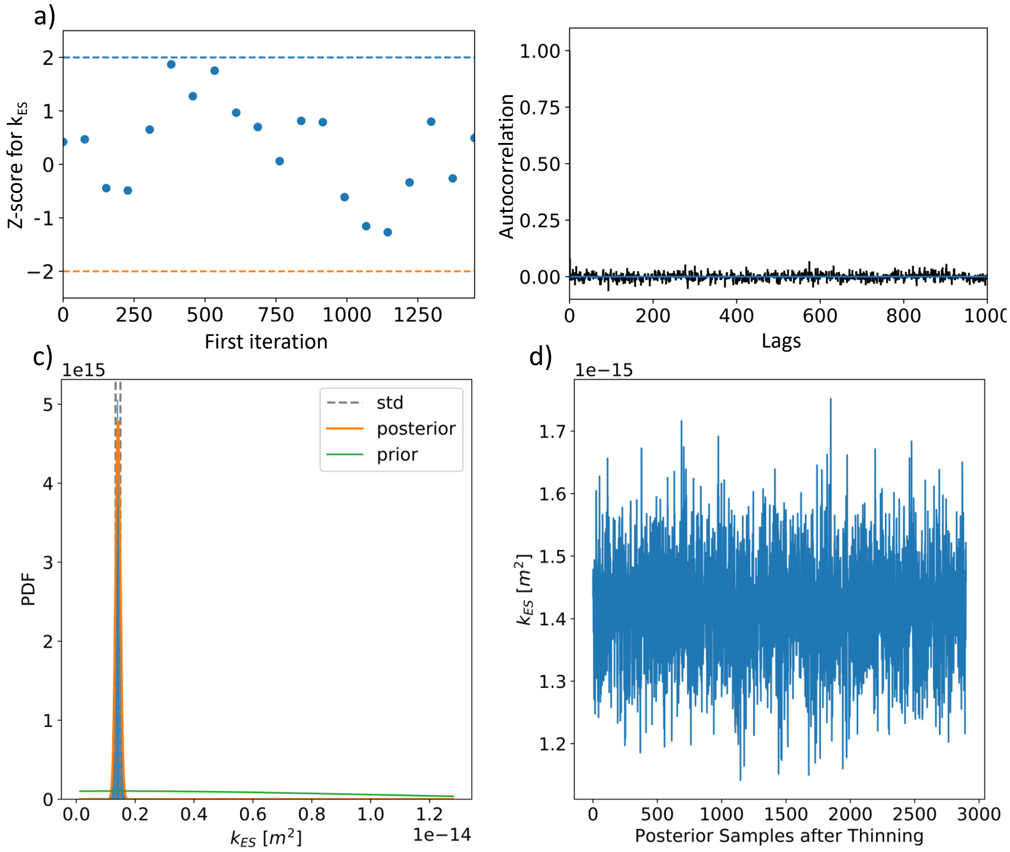

Fig. S1. Posterior analysis of the permeability of the Elbe sandstone I layer (k_{ES}). Shown are the a) Geweke Plot, b) autocorrelation, c) posterior parameter distributions, and d) the trace.

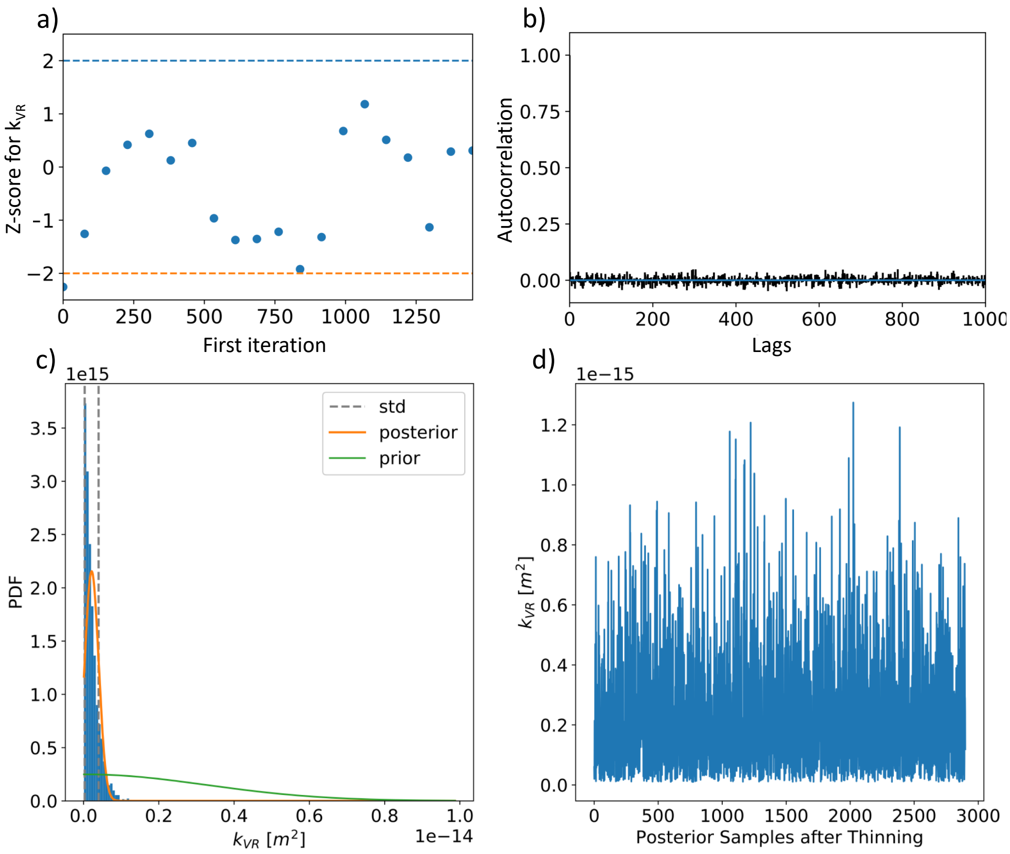

Fig. S2. Posterior analysis of the permeability of the Volcanic rocks (k_{VR}). Shown are the a) Geweke Plot, b) autocorrelation, c) posterior parameter distributions, and d) the trace.

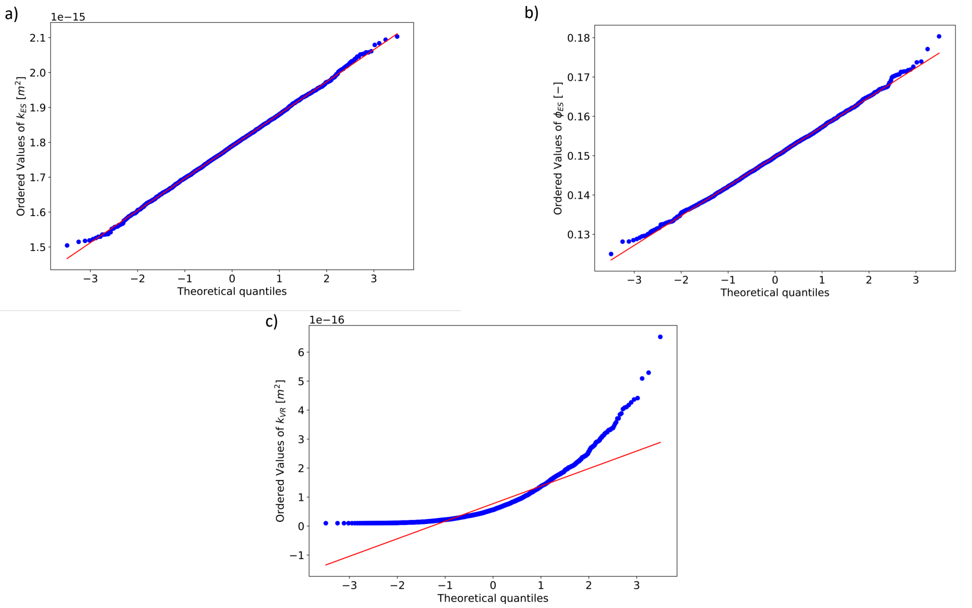

Fig. S3. Quantile-Quantile plots for all hydraulic parameters considered in the uncertainty quantification for the monitoring well E GrSk 3_90. Note that k denotes the permeability, ϕ the porosity, and the subscripts ES and VR the Elbe sandstone I layer, and the Volcanic rocks, respectively.
